# Translating primary care to telehealth: analysis of in-person consultations on diabetes and cardiovascular disease

**DOI:** 10.3399/BJGPO.2022.0123

**Published:** 2023-01-25

**Authors:** Jared Lane, Katrina David, Jayashanthi Ramarao, Kanesha Ward, Sunayana Raghuraman, Moomna Waheed, Annie YS Lau

**Affiliations:** 1 Centre for Health Informatics, Australian Institute of Health Innovation, Macquarie University, Sydney, Australia

**Keywords:** general practice, telehealth, primary healthcare, COVID-19, diabetes mellitus, type 2, cardiovascular diseases

## Abstract

**Background:**

The COVID-19 pandemic had a considerable impact on primary care, resulting in rapid uptake of telehealth. Patients with chronic conditions, such as type 2 diabetes mellitus (T2DM) and cardiovascular disease (CVD), relied heavily on telehealth consultations during this period. It is important to assess whether tasks observed during T2DM or CVD in-person consultations are translatable to telehealth.

**Aim:**

To explore the extent to which in-person GP consultations are translatable to telehealth for patients with T2DM or CVD.

**Design & setting:**

This study screened 281 GP consultations conducted in 2017 within the UK general practice setting for consultations pertaining to T2DM or CVD. Seventeen in-person consultations (in deidentified video and transcript) were selected for further analysis.

**Method:**

Detailed reporting of tasks, physical artefacts, and physical examinations observed during in-person GP consultations. A new scoring method, applying two key metrics, supporting definitions and examples, was designed to assess translatability of clinical tasks to telehealth.

**Results:**

Across the 17 T2DM or CVD in-person consultations analysed, 23 clinical tasks, 21 physical artefacts, and nine physical examinations were observed. Sixty per cent of tasks analysed were deemed either easily or relatively easily translatable to telehealth. Twenty-six per cent of tasks were rated as ‘moderately translatable to telehealth’ but may require a patient obtaining their own equipment. Thirteen per cent of tasks were rated as ‘potentially translatable to telehealth’. No clinical tasks for these cohorts were rated as untranslatable to telehealth.

**Conclusion:**

The majority of tasks observed during T2DM or CVD in-person GP consultations are translatable to telehealth. Further research is warranted to investigate emergent safety concerns from increased uptake of telehealth.

## How this fits in

There is a growing body of work evaluating the effectiveness of telehealth as a modality for primary health care. There are numerous studies on patient and clinician attitudes, and growing evidence that telehealth is effective for managing T2DM and CVD. However, there are no prior studies, to the authors' knowledge, on how translatable tasks observed during in-person primary care consultations are to telehealth. This study aimed to determine the translatability of telehealth for clinical tasks performed for these cohorts.

## Introduction

Before the COVID-19 pandemic, telehealth accounted for only a fraction of GP encounters.^1,2^ As a result of the pandemic, many nations enacted policy responses to expand the eligibility and financial viability of telehealth services,^3,4^ resulting in a worldwide surge in the delivery of GP consultations via telehealth.^5,6^ This rapid shift towards global telehealth adoption means it is difficult to ascertain whether in-person aspects of consultations have been adequately substituted in telehealth.

Patients with chronic diseases require regular contact with GPs for the management of their conditions.^7^ This includes performing a variety of clinical tasks including blood pressure measurement, foot examinations, prescribing medication, and pathology test referrals.”^8,9^ During the pandemic, many have had to use telehealth to maintain continuity of care. However, it is unclear if in-person chronic disease management visits in primary care are indeed translatable to telehealth settings. Patients with T2DM and CVD require extra precaution because they are at high risk of developing COVID-related complications.^10^ While T2DM and CVD are distinct diseases, they are closely linked because they share common risk factors, and people with diabetes are at increased risk of developing CVD.^7^ It is vital to ascertain whether telehealth is an adequate substitute for tasks observed during in-person consultations for T2DM or CVD management in primary care.

Previous studies have demonstrated the effectiveness of telehealth for managing patients with heart failure^11^ and hypertension.^12,13^ Multiple systematic reviews show that telehealth can improve the control of blood glucose levels and other health outcomes for patients with T2DM.^14,15^ Other studies have demonstrated the importance of effective communication in the absence of physical interactions.^16^ Some researchers are investigating the impact of telehealth by analysing primary care system usage before and during COVID-19.^17^ However, no studies to the authors' knowledge, have investigated the extent to which tasks observed during in-person GP consultations are ‘translatable’ to telehealth. This study — focusing on UK in-person GP consultations, and concerning T2DM or CVD — aimed to identify clinical tasks, physical examinations, and physical artefacts utilised during in-person GP consultations, and analyse whether in-person aspects of a consultation are translatable to telehealth. With telehealth set to become a mainstay in health service delivery, this study has important implications for next-generation design of virtual care in primary care settings.

## Method

### Study design

A detailed secondary analysis was conducted of videos recorded in GP consultations, originating from an NHS-ethical approved project, *Harnessing resources from the internet to maximise outcomes for GP consultations (HaRI): a mixed qualitative methods study,* to investigate internet use in general practice. The HaRI study video-recorded 281 consultations from 10 GPs working at eight GP surgeries during 2017, across London and the home counties. See Ethical approval section for details.

### Data collection

Two hundred and eighty-one HaRI video recordings and transcripts were passed through inclusion and exclusion criteria to restrict the analysis to consultations pertaining to T2DM or CVD (See Supplementary Table S1). Two researchers (KD, JR) independently read each GP consultation transcript, and coded types of chronic diseases and presenting issues using NVivo software (version 12). Twenty-one relevant transcripts were identified, but four of the 21 were removed owing to problems with video data, leaving 17 (nine T2DM and eight CVD) consultations for final analysis (see flowchart in Supplementary Figure S1).

#### Deidentification

To maintain privacy of patients and health personnel, a custom-made software comprised of a low-pass filter was developed in-house to blur the faces of individuals in the videos. Thus, all individuals and their faces were blurred and deidentified before any analysis.

### Data analysis

#### Descriptive analysis

Descriptive statistical analysis was applied to patient and consultation characteristics extracted from relevant transcripts.

#### Video and transcript analysis

Two researchers (KD, JR) independently read all 17 transcripts and watched all 17 videos, and extracted any mention of physical artefacts or physical examinations in the transcripts and/or videos, and recorded them for analysis. In all instances where clinical tasks, physical examinations, or physical artefacts were performed or identified, matching transcript excerpts were extracted and recorded within an Excel spreadsheet, allocated to categories inductively formulated during the data-extraction process. These categories included the following:

1. tasks performed during in-person consultations (for example, prescribing medication);
2. physical examinations (for example, auscultation) performed during in-person consultations;
3. physical artefacts (for example, stethoscope) identified during in-person consultations.

#### Data verification

A third researcher (JL) analysed all videos and transcripts to verify extracted information from the initial analysis. All information regarding tasks, physical examinations, and physical artefacts were re-extracted, cross-checked against the initial analysis, and amended as required. In addition, physical artefacts were further categorised into three groups:

- physical artefacts that are readily found in patient’s home setting (for example, computer);
- physical artefacts that are easily acquired through purchase or provision (for example, thermometer);
- physical artefacts that are not easily acquired by patients in the community (for example, stethoscope).

Personal items, such as mobile phones, were excluded from the physical artefact list, unless they were used to support a task (for example, Fitbit used to support a discussion about exercise).

#### Time analysis

One researcher (JL) reanalysed all 17 videos and extracted the following information: length of entire consultation; number of physical examinations per consultation; and length of time for physical examination(s). These analyses were also performed independently for each condition (T2DM and CVD).

#### Translatability to telehealth analysis

A scoring system was developed to rate the ‘translatability’ of in-person GP tasks to telehealth. This was partly adapted from a study by Croymans *et al* who developed a rating scale to rank the appropriateness of certain conditions to telehealth.^18^ Rather than ranking conditions, the research aimed to develop a rating system for individual tasks performed during consultations. Developing the scoring system involved analysing deidentified consultation videos, discussions between researchers (JL, KW, SR), and reviewing relevant literature. It became apparent that tasks requiring specialised equipment or in-person expertise, for example, physical examinations, were the key factors in how readily tasks could be replicated using telehealth. These discussions and analyses led to the development of the following scoring system procedure:

- **Step 1**: Assess the extent to which a task requires ‘clinical endorsement’, based on a 5-point score (Table 1, metric 1).
- **Step 2**: Assess the extent to which a task requires ‘physical artefacts or physical interaction’, based on a 5-point score (Table 1, metric 2).
- **Step 3**: Sum up the scores from steps 1 and 2 to calculate an overall score out of 10 that describes how well this task can be translated to telehealth, that is, 'translatability to telehealth score' (see Table 2).
- **Step 4:** Categorise the type of 'virtual care solution' proposed for this task, based on the 10-point score from step 3, according to rules defined in Table 3.

**Table 1.** Metrics used to score translatability of in-person tasks

**Metric 1: Clinical endorsement score**	
**Score**	**Description**
1/5	Requires in-person clinical expertise; for example, swabs, smears, excising lesions, giving injections
2/5	In-person clinical expertise is preferred but remote digital solutions are possible; for example, skin inspections, auscultation, palpation, foot examinations
3/5	Clinical endorsement is required for interpretation of results that the patient can collect in their homes, as well as for tasks that have current digital solutions; for example, temperature checks, weight, blood pressure, glucose readings, oxygen saturation, heart rate
4/5	Medical endorsement is required for tasks, such as targeted history-taking, but no specific equipment is required, making it easier to perform over telehealth
5/5	Medical expertise may not necessarily be required to complete this task; for example, printing
**Metric 2: Physical artefacts or physical interactions score**	
**Score**	**Description**
1/5	Requires equipment or physical examination in a manner not translatable to telehealth; for example, swabs, smears and so on
2/5	Requires equipment or physical examination potentially translatable to telehealth but preferable in-person; for example, auscultation, physical inspection involving palpation
3/5	Requires equipment that is easily purchasable in most pharmacies, or requires pick-up or delivery; for example, thermometers, blood pressure monitors
4/5	Requires equipment that is easily accessible in a patient’s home, and thus can be translated over telehealth; for example, computer, printer, weight scale
5/5	Does not require any equipment, thus readily translatable over telehealth; for example, discussing diet or medication use

**Table 2.** Translatability to telehealth score interpretation. *A non-colour dependent version of this table is available in the supplementary materials.*

**Metric 1: Clinical endorsement score**	Metric 2: Physical artefacts or physical interactions score				
	5	4	3	2	1
**5**	10	9	8	7	6*
**4**	9	8	7	6	5*
**3**	8	7	6	5	4*
**2**	7	6	5	4	3*
**1**	6*	5*	4*	3*	2*

*Not currently translatable to telehealth. Careful attention required when evaluating whether a task (with this score combination) is indeed translatable using current forms of technology.

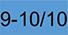
: Easily translatable over telehealth with no additional physical artefacts required = type 5.

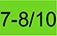
: Relatively easy to translate over telehealth, with minimal but easily accessible equipment required = type 4.

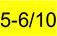
: Moderately translatable over telehealth but may require patient to acquire their own equipment to do so = type 3.

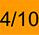
: Has the potential to be translated over telehealth but may require clinician to administer virtual examination, and may require patient to obtain special equipment and training = type 2.

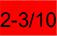
: Not amenable to being replicated over telehealth at this stage = type 1.

**Table 3.** Translatability to telehealth score and corresponding virtual care solution type

Translatability to telehealth score	Description	Virtual care solution	Description
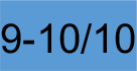 = Type 5	Easily translatable over telehealth with no additional physical artefacts required	Type 5	Clinicians and/or patients can easily exchange information over the telephone and/or video (for example, discussing diet or medication)
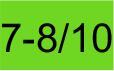 = Type 4	Relatively easy to translate over telehealth, with minimal but easily accessible equipment required	Type 4	Patients conduct self-assessment at home and communicate self-reported findings; for example, measuring weight, print electronic requests or results
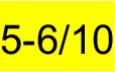 = Type 3	Moderately translatable over telehealth but may require patient to acquire their own equipment to do so	Type 3	Patients acquire necessary artefacts through purchase or pick-up, and perform and communicate findings. Virtual guidance or training may be required; for example, measuring oxygen saturation or blood pressure
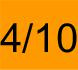 = Type 2	Has the potential to be translated over telehealth but may require clinician to administer virtual examination, and may require patient to obtain special equipment and training	Type 2	Clinician administers virtual examination. May require patient to obtain special equipment and training; for example, virtual foot examination
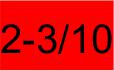 = Type 1	Not amenable to being replicated over telehealth at this stage	N/A	N/A

Table 1 describes the metrics from steps 1 and 2 and includes definitions and examples for each point-score for each of the key domains. These descriptions are used as a guide to score each task identified during in-person GP consultations. See Supplementary Table S2 for scoring-system rationale for different task-types.

Table 2 illustrates how the two key metrics are combined to calculate the ‘translatability to telehealth’ score out of 10.

Table 3 shows how the scores from Table 2 are further categorised into five ‘types’, corresponding to appropriate virtual care solutions, which are designated as type 1 through to type 5.

## Results

### Patient and consultation characteristics

Overall, 17 T2DM or CVD in-person consultations were analysed in this study, where nine pertained to patients with T2DM and eight pertained to patients with CVD. Refer to Supplementary Table S3 for patient and consultation characteristics.

### Physical examinations performed during in-person consultations

**Figure 1.**
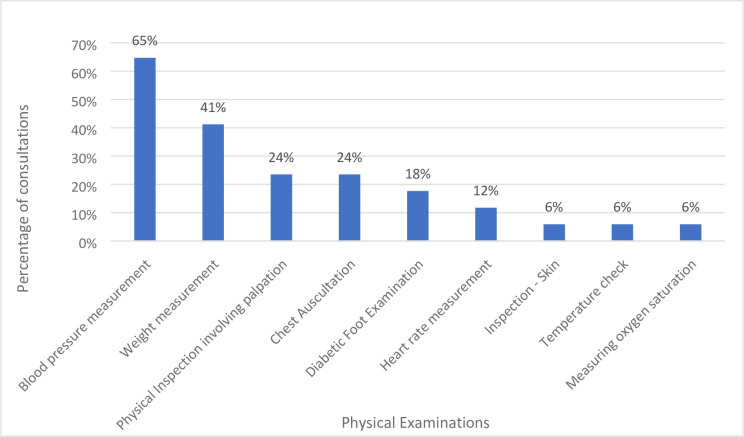
Frequency of physical examinations performed during in-person consultations, as a percentage of total consultations (*n* = 17)

Figure 1 shows the frequency of physical examinations across observed consultations. Overall, nine physical examinations were conducted across these 17 in-person consultations. Eighty-eight per cent (*n* = 15/17) of consultations featured physical examinations.

#### Time analysis of physical examination(s) during consultation

Thirty-three physical examinations were observed across these 17 in-person consultations. The average total length of GP consultations was 13 minutes 02 seconds. The average time spent on physical examinations(s) during a consultation was 2 minutes 26 seconds. The average time taken for physical examinations during consultations was 1 minute and 55 seconds for patients with T2DM, and 2 minutes and 53 seconds for patients with CVD. On average, 22% of total consultation time is devoted to physical examination(s) and 78% involves no physical examination. Refer to Supplementary Table S4.

### Physical artefacts used during in-person consultations

**Figure 2.**
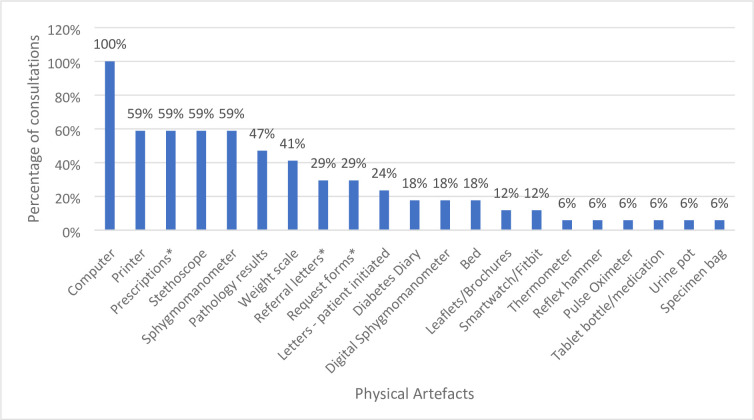
Frequency of physical artefacts used during in-person consultations (*n* = 17). *Physical artefact dispensed either by GP or at GP reception desk

Figure 2 shows the frequency of physical artefacts observed across in-person T2DM or CVD consultations. Overall, 21 physical artefacts (for example, computer, stethoscope) were identified across these 17 consultations. Of the 21 physical artefacts observed, 12 were defined as readily found in a patient’s home setting, six were defined as easily acquired through purchase or provision, and three were defined as not easily acquired (Supplementary Tables S5, S6, and S7).

### Tasks performed during in-person consultations

**Figure 3.**
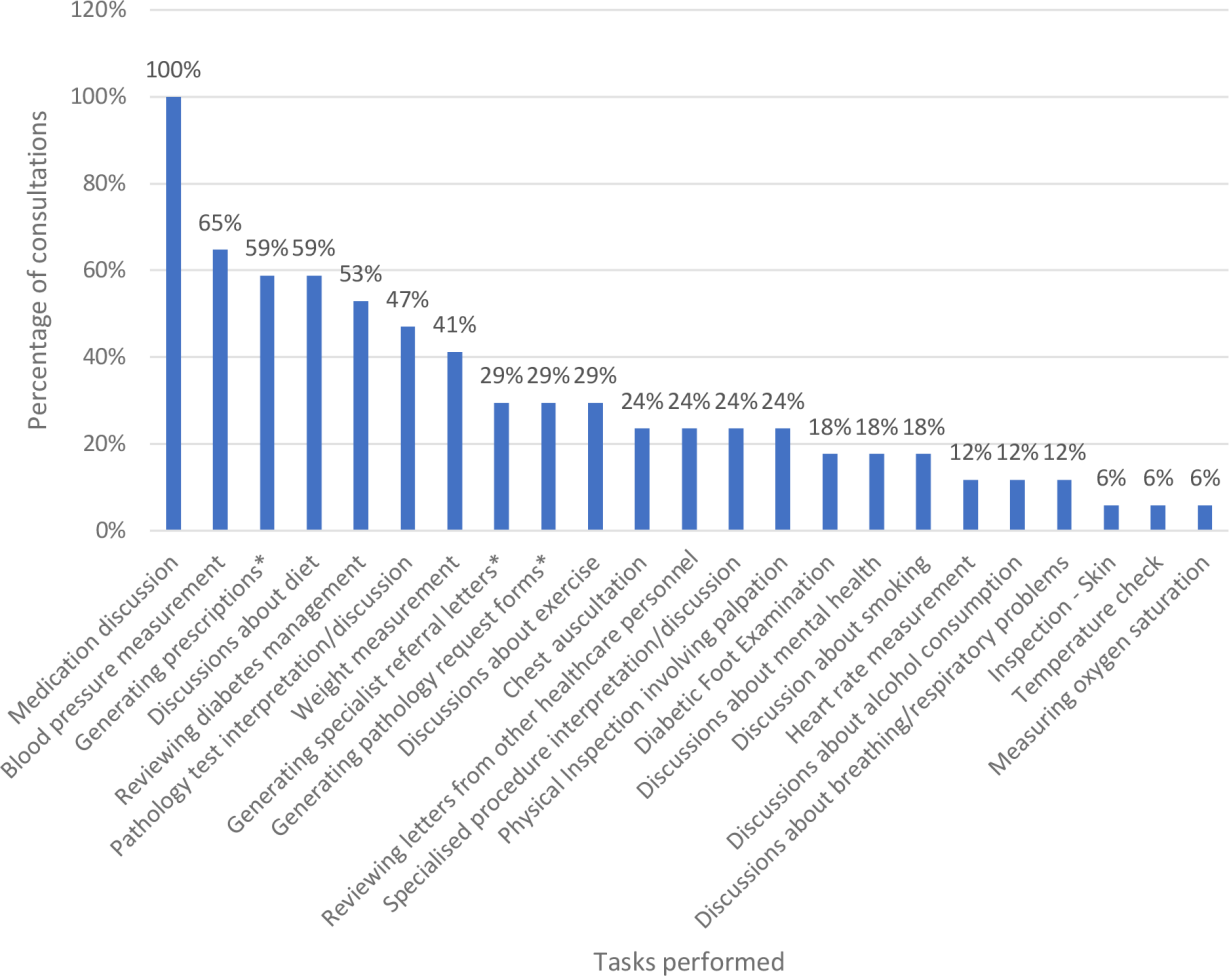
Tasks performed during in-person T2DM or CVD GP consultations as a percentage of total consultations, ordered by frequency (*n* = 17). ^*^Physical dispensation either by GP or at GP reception desk

Figure 3 outlines the frequency of tasks observed across T2DM or CVD in-person consultations. A total of 23 tasks were observed across 17 consultations. Out of the 23 tasks performed, 39% (*n* = 9/23) involved physical examination, and 74% (*n* = 17/23) of these tasks required physical artefacts at least some or all of the time.

#### Translatability of tasks to telehealth

Supplementary Table S8 describes how translatable these 23 clinical tasks are to telehealth across T2DM and CVD consultations. Across the 23 tasks, the average score for telehealth metric was as follows:

- clinical endorsement was 3.4/5 (where score 1 = ‘Requires in-person clinical expertise’, 5 = ‘Clinical expertise is not necessarily required to complete this task’);
- physical artefacts or physical interactions was 3.8/5 (where score 1 = ‘Requires equipment or physical examination in a manner not translatable to telehealth’, 5 = ‘Does not require any equipment, thus readily translatable over telehealth’); and
- translatability to telehealth was 7.2/10 (where score 1 = ‘Not amenable to being replicated over telehealth at this stage’, 10 = ‘Easily translatable over telehealth with no additional physical artefacts required’).

Regarding telehealth metric of virtual care solution types, all 23 tasks observed across T2DM or CVD consultations were deemed translatable or potentially translatable to telehealth. Overall, the following was found:

- no tasks were rated as type 1 (‘Not amenable to being replicated over telehealth at this stage’);
- 13% (*n* = 3/23) of tasks were rated as type 2 (‘Has the potential to be translated over telehealth but may require clinician to administer virtual examination, and may require patient to obtain special equipment and training’); for example: chest auscultation;
- 26% (*n* = 6/23) were rated as type 3 (‘Moderately translatable over telehealth but may require patient to acquire their own equipment’); for example, measuring oxygen saturation;
- 30% (*n* = 7/23) were rated as type 4 (‘Relatively easy to translate over telehealth, with minimal but easily accessible equipment required’); for example, measuring weight;
- 30% (*n* = 7/23) were rated as type 5 (‘Easily translatable over telehealth with no additional physical artefacts required’); for example, discussion about exercise.

## Discussion

### Summary

The average translatability to telehealth score was 7.2 out of 10, indicating that overall, tasks for T2DM or CVD cohorts were ‘relatively easy to translate over telehealth, with minimal but easily accessible equipment required’. The findings revealed that 78% of consultation time for these cohorts is devoted to tasks that involve no physical examination. While 88% of consultations involved one or more physical examination(s), most of these physical examinations require equipment that can be easily acquired, and results communicated remotely for medical interpretation. Analysis of physical artefacts revealed that 85% (*n* = 18/21) of physical artefacts are either readily available in home settings, or easily acquired through purchase or provision.

The ‘translatability to telehealth’ scoring system found that while on average tasks were rated as easily translatable, certain important tasks are not easily translated to telehealth at this time. This includes certain essential tasks; for example, chest auscultation. Categorising tasks and virtual care solutions is useful for identifying the gaps that still exist in telehealth and where future research into digital solutions is required, if telehealth is to have a role in primary care delivery in the long run.

### Strengths and limitations

The major strength of this study was that it analysed actual GP consultation data via video and transcript, rather than using self-report data such as survey and interview. This objective analysis permits less capacity for recall bias and measurement error commonly associated with self-report methods. This study involved analysis of consultations across multiple GPs and GP clinics, thereby reducing potential clinician bias.

These findings were limited to a sample size of 17 consultations and low sample size could result in an incomplete understanding of the scope of clinical tasks for these cohorts. All consultations occurred in the UK and were conducted in English, potentially limiting generalisability of the findings to other contexts (for example, non-English-speaking countries, or other healthcare systems). Further research using larger datasets and different settings would be useful to identify any differences in disease management for these cohorts. Finally, this study could not analyse unique patient factors, for example, digital literacy, cognitive capacity, which impact on the effectiveness of telehealth. Future research should include this patient context when analysing the translatability of clinical tasks to telehealth.

### Comparison with existing literature

This HaRI dataset was used by Stevenson *et al* to analyse how the internet was used during GP consultations.^19^ The method used in this study for scoring translatability of tasks to telehealth is a novel approach, to the authors' knowledge. The appropriateness scale, developed by Croymans *et al,* scored ‘diabetes management’ 6.5/9, indicating it was appropriate for telehealth,^18^ and ‘High blood pressure management’ skewed marginally towards ‘appropriate’ with a score of 5.6/9.^18^ The findings were consistent with other studies analysing telehealth usage for patients with CVD or T2DM.^11–15^

Scoring certain complex tasks as ‘potentially translatable to telehealth’ is consistent with emerging evidence on virtual care solutions. There is existing guidance for performing remote physical assessments including foot examinations,^20^ musculoskeletal examinations,^21^ as well as chest auscultation using Bluetooth-connected electronic stethoscopes.^22^

### Implications for research and practice

There are safety concerns arising from increased uptake of remote examination. A lack of clinical expertise and an inability to verify the accuracy of home-based equipment could result in measurement error. Measurement errors could lead to misdiagnoses and inappropriate treatment. Greater research is needed on the safety aspects of home monitoring and remote examination.

Certain clinical tasks, such as chest auscultation, are not readily amenable to telehealth at this time. Further research is needed to evaluate the viability of existing digital solutions for these tasks, as well as the development of new technologies for these complex tasks.

There is a risk that telehealth could create ‘low-value care’; that is, care that is either ineffective, inefficient, or unwanted.^23^ If telehealth patients still require in-person consultations afterwards, this would be inefficient because it would increase the total amount of care in the system. Future research could build on the ‘translatability to telehealth’ scoring system to develop a more robust telehealth triage protocol.

Telehealth is a tool with the ability to either reduce health inequities through increased access to care, or to widen inequities through digital poverty. Future research is needed to identify how barriers to the adoption of telehealth can be eliminated, as well as identifying strategies to maximise the positive potential of this technology.

It is likely that a hybrid care model, incorporating both in-person and telehealth consultations into routine care, will be commonplace moving forward. Further research is required evaluating the safety and health outcomes for patients undergoing hybrid care.

## Data

The data relied on in this study are not publicly available due to their containing information that could compromise the privacy of research participants.
